# A Narrative Review of the Applications of Ex-vivo Human Liver Perfusion

**DOI:** 10.7759/cureus.34804

**Published:** 2023-02-09

**Authors:** Trisha Kanani, John Isherwood, Eyad Issa, Wen Y Chung, Matteo Ravaioli, Marco R Oggioni, Giuseppe Garcea, Ashley Dennison

**Affiliations:** 1 Department of Hepato-Pancreato-Biliary Surgery, University Hospitals of Leicester NHS Trust, Leicester, GBR; 2 Department of Medical and Surgical Sciences, University of Bologna, Bologna, ITA; 3 Department of Genetics and Genome Biology, University of Leicester, Leicester, GBR

**Keywords:** liver, human liver perfusion, liver perfusion, organ preservation, normothermic, ex-vivo, human liver, perfusion

## Abstract

Ex-vivo perfusion describes the extra-corporeal delivery of fluid to an organ or tissue. Although it has been widely studied in the context of organ preservation and transplantation, it has also proven to be an invaluable tool in the development of novel models for translational pre-clinical research. Here, we review the literature reporting ex-vivo human liver perfusion experiments to further understand current perfusion techniques and protocols together with their applications. A computerised search was made of Ovid, MEDLINE, and Embase using the search words “ex-vivo liver or hepatic perfusion”. All relevant studies in English describing experiments using ex-vivo perfusion of human livers between 2016 and 2021, inclusive, were included. Of 21 reviewed studies, 19 used ex-vivo human liver perfusion in the context of allogeneic liver transplantation. The quality and size of the studies varied considerably. Human liver perfusion was almost exclusively limited to whole organs and “split” livers, although one study did describe the successful perfusion of tissue sections following a partial hepatectomy. This review of recent literature involving ex-vivo human liver perfusion demonstrates that the technique is not limited to whole liver perfusion. Split-liver perfusion is extremely valuable allowing one lobe to act as a control and increasing the number available for research. This review also highlights the present lack of any reports of segmental liver perfusion. The discarded donor liver is a scarce resource, and the successful use of segmental perfusion has the potential to expand the available experimental models to facilitate pre-clinical experimentation.

## Introduction and background

Ex-vivo perfusion describes the extra-corporeal delivery of fluid to an organ or tissue, usually via the lymphatic or circulatory system. The aim is to maintain the physiology and metabolism of the organ as close as possible to the in-vivo situation by the appropriate delivery of oxygen, nutrients, and pharmacological agents. Ex-vivo machine perfusion was first described in 1935 by Alexis Carrel and Charles Lindbergh in “The Culture of Organs”, where they reported the successful perfusion of the feline thyroid gland for up to 21 days [1]. Subsequently, ex-vivo machine perfusion has been widely studied as a means of organ preservation, particularly in the context of transplantation of organs, including the lung, liver, and kidneys [2-4].

The liver has a dual blood supply with approximately two-thirds of hepatic blood flow being supplied by the portal vein (PV) and the remainder via the hepatic artery (HA) [5]. For this reason, ex-vivo hepatic perfusion usually involves perfusion via the PV, with or without perfusion via the HA. The perfusion circuit includes a chamber containing the organ with the outflow being collected following cannulation (a closed circuit) or free drainage. After collection, effluent blood from the organ is held in a systemic venous reservoir and subsequently oxygenated and pumped via a high-speed motor device into the PV and/or HA depending on the nature of the study. Separate pressure regulators or a portal venous reservoir with an outflow controller are employed to ensure a lower perfusion pressure for the PV.

This technique has been employed and examined at a variety of temperatures with the most common being normothermic machine perfusion (NMP) and hypothermic machine perfusion (HMP). NMP aims to mimic physiological conditions as closely as possible and is usually conducted at 34-38°C with an oxygenated perfusate to support normal cellular metabolism [6]. HMP is usually performed at 4-8°C and consequently does not require oxygenation of the perfusate [6]. There are a number of commercially available machine perfusion devices in clinical use currently, including the “Liver Assist^TM^” (Organ Assist, Groningen, The Netherlands) and the portable “OrganOx metra^TM^” (OrganOx Ltd, Oxford, UK). The “Liver Assist^TM^” facilitates NMP and HMP whereas the “OrganOx metra^TM^” only permits NMP [7]. “VitaSmart^TM^” by Bridge to Life Ltd. (Northbrook, United States of America) is another multi-organ machine perfusion system currently being investigated in the USA in a randomised clinical trial of ischaemia reperfusion treatment using portal venous hypothermic oxygenated perfusion [8,9].

The first clinical series of HMP of human livers prior to transplantation was reported in 2010 by Guarrera et al. [10]. Twenty grafts were perfused at 4-6°C without oxygenation of the perfusate. This pilot case-controlled series compared HMP-preserved transplanted livers with those preserved by the traditional technique of static cold storage (SCS) at 0-4°C and demonstrated its safety and potential to improve graft function and reduce the consequences of ischaemia-reperfusion injury (IRI).

The first NMP of human livers was described in 2013 by op den Dries et al. [11]. They studied the feasibility of NMP in four discarded donor livers. The PV and HA were perfused for six hours at 37°C resulting in improvements in lactate clearance, bile production, and histology that would be expected with a viable and functioning organ. The benefits of NMP over SCS and HMP were the optimisation of the graft, the ability to assess the viability and function more accurately prior to transplantation, and the potential in the clinical setting of increasing the available donor pool [11].

Although ex-vivo perfusion of human organs has been widely studied in the context of organ preservation and transplantation, it has also been shown to be an invaluable tool in the development of novel models for translational pre-clinical research [12,13]. These models represent a far more ethical alternative to live animal experimentation, which allows the more accurate examination of human organ responses to noxious external stimuli. Importantly, due to the isolated nature of the organ, they also facilitate detailed examination of the response to the change of a single parameter or selected multiple parameters without confounding systemic responses. For example, a recent study by Carreno et al. utilised an ex-vivo human spleen perfusion model to study the response of human splenic macrophages to pneumococcal infection [12]. Following splenectomy, human spleens underwent NMP with a haemoglobin-based oxygen carrier. The use of NMP to develop a model of infection led to the novel demonstration that the spleen and not the lung is the most likely source of bacteraemia during systemic infection from pneumococcal pneumonia demonstrating the value of ex-vivo perfusion studies beyond transplantation [12].

Here, we summarise the recent literature describing ex-vivo perfusion experiments in the human liver to review current perfusion techniques and applications of ex-vivo human liver perfusion.

## Review

**Figure 1:**
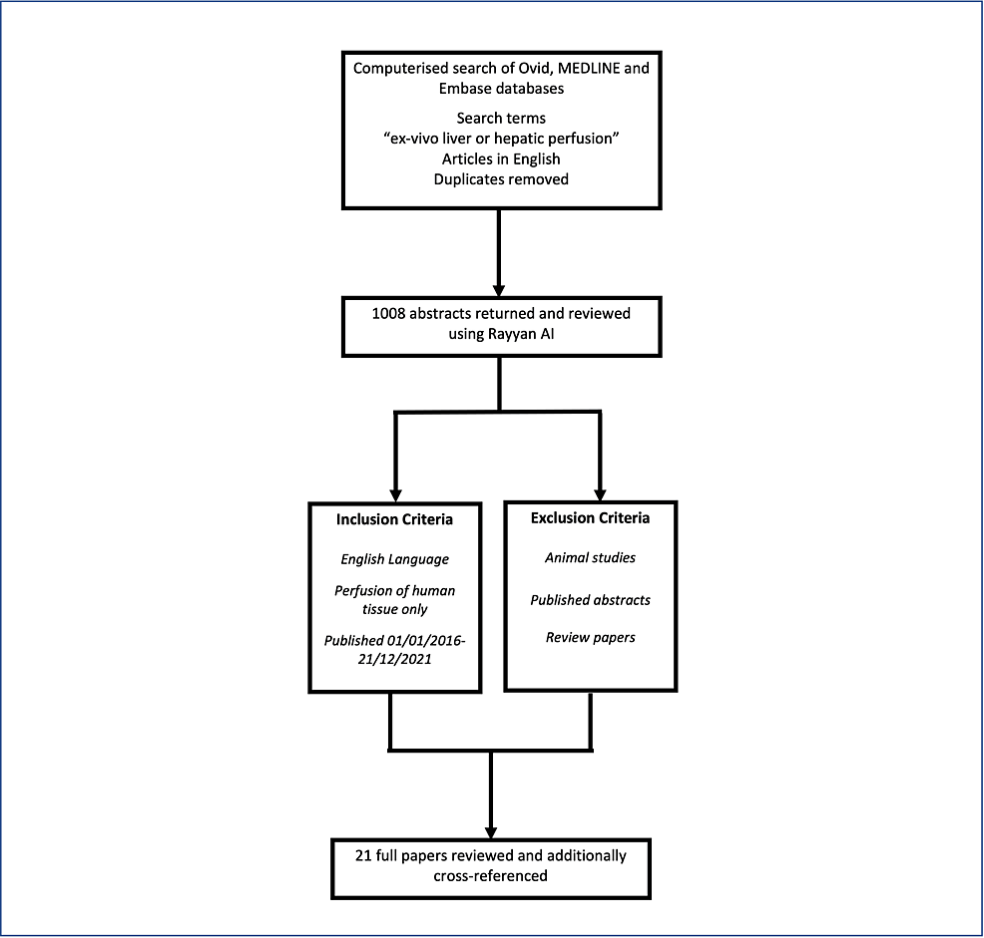
The search strategy

This review examines the current literature on experimental studies involving ex-vivo perfusion of human livers and their applications. A computerised search was made of Ovid, MEDLINE and Embase using the search words “ex-vivo liver or hepatic perfusion”. All duplicate studies were removed and 1008 abstracts in the English language were examined using the web-based application Rayyan AI [14]. All relevant studies describing experiments using ex-vivo perfusion of human livers between 01/01/2016 and 31/12/2021 were included. Published abstracts were excluded. A total of 21 papers describing experimental studies of the ex-vivo perfusion of human livers within this time period were reviewed and additionally cross-referenced. Figure 1 summarises the search strategy.

Current literature

Table 1 summarises the literature reviewed. Of the 21 papers reviewed, 19 reported ex-vivo human liver perfusion in the context of allogeneic liver transplantation. Only two of the studies in Table 1 describe non-allotransplantation applications for ex-vivo human liver perfusion. The quality and size of the studies varied considerably, from simple case reports to a large, randomised trial describing 137 ex-vivo human liver perfusions (Table 1).

**Table 1:** Results of the literature search Studies included as part of the literature search with citations: [15-35].

Authors	Citation	Year	Context	Study design	The portion of the liver perfused	Number of perfusions
Schreiter et al.	[15]	2016	Hepatotoxicity	Cohort study	Tissue section	9
Kong et al.	[16]	2019	Operative technique	Case reports	Whole liver	2
Matton et al.	[17]	2020	Transplantation	Cohort study	Whole liver	12
Bral et al.	[18]	2017	Transplantation	Clinical trial	Whole liver	9
Nasralla et al.	[19]	2018	Transplantation	Clinical trial	Whole liver	137
Selzner et al.	[20]	2016	Transplantation	Clinical trial	Whole liver	10
Ghinolfi et al.	[21]	2019	Transplantation	Clinical trial	Whole liver	10
Ravaioli et al.	[22]	2020	Transplantation	Clinical trial	Whole liver	10
van Rijn et al.	[23]	2017	Transplantation	Cohort study	Whole liver	10
van Rijn et al.	[24]	2021	Transplantation	Clinical trial	Whole liver	78
Hoyer et al.	[25]	2016	Transplantation	Case series	Whole liver	6
Goumard et al.	[26]	2020	Transplantation	Qualitative	Whole liver	7
Bruinsma et al.	[27]	2016	Transplantation	Cohort study	Whole liver	21
Huang et al.	[28]	2020	Transplantation	Pre-clinical trial	Split liver	22
Lau et al.	[29]	2021	Transplantation	Case reports	Split liver	2
Thorne et al.	[30]	2021	Transplantation	Case report	Split liver	1
Haque et al.	[31]	2021	Transplantation	Pre-clinical trial	Whole + split liver	5
Maroni et al.	[32]	2021	Transplantation	Cohort study	Whole liver	19
Ciria et al.	[33]	2019	Transplantation	Pre-clinical study	Whole liver	4
Zhao et al.	[34]	2018	Transplantation	Case reports	Whole liver	2
Westerkamp et al.	[35]	2016	Transplantation	Pre-clinical trial	Whole liver	18

Schreiter et al. are the only group to describe ex-vivo human liver perfusion as a model for liver disease [15]. Nine sections of human liver tissue (six as a control) underwent NMP for up to 30 hours to study acetaminophen-induced liver injury. Following incubation with low-dose acetaminophen, the sections exhibited reduced function and viability with a lower indocyanine green half-life and hepatocyte damage demonstrated on histological examination of biopsies. They suggested that the model would allow dose-dependent hepatotoxicity investigation in human liver tissue, which more closely resembles human in-vivo physiology in contrast to cell culture and animal models [15].

Kong et al. describe two case reports of ex-vivo liver resection and auto-transplantation (ERAT) using a novel surgical technique [16]. ERAT is a technique used in the treatment of end-stage hepatic alveolar echinococcosis (HAE). Once the liver is retrieved, it is crucial that ex-vivo perfusion is initiated promptly to minimise the warm ischaemic (WI) time and, consequently, hepatocyte injury [16]. In severe HAE this can prove challenging, particularly where there is an invasion of the porta hepatis and secondary cavernous transformation of the portal vein making it difficult to find a suitable inflow vessel to perfuse the liver [16]. Kong et al. described catheterisation of the transhepatic-intrahepatic branches of the PV to allow prompt perfusion at 0-4°C with Custodiol^TM^ (Dr. Franz Köhler Chemie GmbH, Bensheim, Germany) preservation solution following retrieval. Both cases had a warm ischaemia time of only two minutes and underwent successful ERAT despite severe thrombosis and obstruction of the portal venous branches [16].

The remainder of the studies reviewed describes clinical trials, case reports or series, and experiments designed to study the optimisation of liver preservation technique and examine markers of viability in the context of liver allotransplantation. There is a well-documented shortage of liver donors mandating the use of extended criteria donors (ECD) to expand the donor pool. ECD include older grafts, those donated after circulatory death (DCD) and fatty livers. As these livers are more vulnerable to IRI, machine perfusion facilitates organ recovery and the opportunity to assess the organ’s viability and hence suitability for transplantation [17].

Normothermic machine perfusion

SCS is the traditional method for liver preservation but has been shown to have higher rates of post-transplantation graft dysfunction, IRI, and ischaemic cholangiopathy when compared with livers preserved with NMP [18]. NMP, which allows viability testing prior to transplantation and drug delivery to aid recovery, occupies an important position when studying the recovery of marginal donor livers and expanding the donor pool.

A Canadian clinical trial by Bral et al. was the first in North America to compare SCS with NMP prior to transplantation [18]. They transplanted nine livers following NMP with a median duration of 11.5 hours (3.3-22.5 hours) and found that when compared with transplanted livers using SCS preservation, there was no statistically significant difference in 30-day mortality, although the NMP group did have a longer length of stay on intensive care and in hospital [18]. This demonstrated the feasibility of NMP prior to transplantation and that larger randomised clinical trials were warranted.

Nasralla et al. compared liver preservation with SCS to NMP in a much larger, multi-centre randomised trial with 220 liver transplantations [19]. They found that organ preservation with NMP resulted in a 50% reduction in graft injury demonstrated with lower peak aspartate aminotransferase (AST) levels during the first seven days following transplantation. There was no significant difference in bile duct complications and patient survival. This trial demonstrated how liver preservation with NMP could improve outcomes following liver transplantation if employed in routine clinical practice [19].

Bral et al. used Gelofusine^TM^ (B. Braun, Melsungen, Germany), which is not approved for clinical use in North America, and type O packed red blood cells as the perfusate [18]. Selzner et al. studied the use of a human albumin-based bovine-derived solution called STEEN Solution^TM^ (XVIVO Perfusion AB, Goteborg, Sweden) in place of Gelofusine^TM^, during NMP on the OrganOx metra^TM^ device [20]. Ten livers underwent NMP with STEEN Solution^TM^ and type O packed red blood cells prior to transplantation into suitable recipients. The outcomes were comparable to patients with transplanted livers preserved by SCS. Although there are no studies directly comparing STEEN Solution^TM^ to Gelofusine^TM^ in NMP, STEEN Solution^TM^ is currently approved for use in lung preservation in Canada and the United States, whereas Gelofusine^TM^ is not available [20].

Ghinolfi et al. examined the outcome following transplantation of livers from donors aged 70 or over perfused by NMP [21]. Older grafts tend to be more steatotic and susceptible to IRI due to poor tolerance to cold ischaemia compared with younger grafts. Ten livers were preserved by SCS and 10 underwent NMP with the Liver Assist^TM^ device prior to transplantation [21]. The NMP group demonstrated histological evidence of reduced IRI compared with the SCS group, highlighting the potential of NMP to expand the donor pool.

Hypothermic machine perfusion

Although traditionally HMP was not employed with oxygenation of the perfusate, the introduction of oxygenation during HMP has now been widely studied. Hypothermic oxygenated perfusion (HOPE) involves oxygenation of the perfusate when the PV is the only vessel perfused. Dual hypothermic oxygenated perfusion (DHOPE) utilises oxygenated fluid at hypothermic temperatures with perfusion of both the PV and HA. The use of oxygenated HMP has been suggested to improve graft function following transplantation by restoring levels of adenosine triphosphate (ATP) [22].

A case-control study reported in 2017 by van Rijn et al. examined the role of end-ischaemic DHOPE in the recovery of DCD livers [23]. Ten livers underwent at least two hours of DHOPE on the Liver Assist^TM^ device immediately prior to transplantation. They were able to show that, in comparison to transplanted livers that were preserved by SCS alone, the DHOPE group demonstrated a reduction in IRI and an improved early graft function. These findings are believed to represent the result of ATP restoration during oxygenated hypothermic perfusion [23].

In 2020, Ravaioli et al. published the results of the Italian non-randomised clinical trial using ECD and HOPE for liver and kidney transplantation [22]. When compared with transplanted livers preserved with SCS alone, HOPE was shown to be a safe technique, which reduced ischaemic preservation injury in liver transplantation, and it was also the first clinical trial to use HOPE in kidney transplantation [22]. Their protocol describes the benefits of graft washing at the end of the ischaemia phase to flush out waste products.

The results of a large, multi-centre, randomised, controlled clinical trial of DHOPE-DCD were published in 2021 by van Rijn et al. [24]. A total of 78 DCD liver grafts underwent DHOPE for two hours on the Liver Assist^TM ^device prior to transplantation. In comparison with a control group of livers transplanted following SCS, DHOPE led to a lower risk of symptomatic non-anastomotic biliary strictures at six months post-transplantation (although this was a large study across six centres, there are limitations due to the follow-up duration of only six months) [24]. This further emphasises the benefits of intra-hepatic ATP restoration with HOPE in reducing cholangiopathy secondary to biliary IRI.

Controlled oxygen re-warming

Controlled oxygenated re-warming (COR) describes a process where the temperature increase between SCS and subsequent perfusion is undertaken in a slower, more controlled manner resulting in a more gradual increase in metabolic activity and a consequent reduction of oxidative injury [24]. The principle underpinning COR is the slow re-warming process, which is essential to ensure organ integrity and maintenance of graft function [24].

Hoyer et al. presented the first clinical series of COR after SCS prior to transplantation in humans [25]. COR was used in six marginal donor livers, which were perfused using the Liver Assist^TM^ device with Custodiol^TM^ preservation solution as the perfusate. The COR protocol involved perfusion at 10°C with a gradual increase of the temperature to 12°C, 16°C, and 20°C after 30, 45, and 60 minutes, respectively, and the grafts were then flushed with a cold solution immediately prior to transplantation [25]. The study demonstrated the feasibility of COR following SCS with all transplant recipients having normal liver function tests after six months of follow-up.

The various temperatures at which human livers should be perfused ex-vivo and the rate of increase remain the subject of debate. It has been argued that combining the various modalities in a dynamic fashion with HOPE or DHOPE, COR and NMP is likely to be the most effective [26]. However, a shift from HMP to NMP is technically challenging if fresh toxin-free perfusate is to be used after the change in conditions as most commercially available circuits do not incorporate an intermediate reservoir to facilitate the change of perfusate. To address this, Goumard et al. developed an adaptable circuit and after performing seven perfusions, they were able to demonstrate that it could support dynamic perfusion by enabling a reservoir change to be performed very quickly (within seconds) without interrupting the perfusion circuit [26].

Novel markers of viability

The introduction of NMP for the preservation of donor livers has allowed the metabolic state and viability of the liver to be assessed dynamically prior to transplantation, which is not possible during SCS. Traditionally, hepatic viability is assessed by measuring bile production, liver enzyme release, lactate clearance, and ATP levels.

Bruinsma et al. described the change in metabolomics for human livers in various states of injury undergoing sub-NMP at 21°C [27]. Crushed biopsies were analysed at various time points using mass spectrometry to measure metabolic cofactors. During machine perfusion, they demonstrated that where livers were found to be undergoing metabolic recovery, their metabolic co-factors and redox shifts were improved. They were also able to show that there was a recovery of deteriorating pathways such as in lactate metabolism induced by ischaemia. Interestingly, metabolic profiling was able to discriminate between livers with severe injury, either steatotic or ischaemic, from those suitable for transplantation [27].

It has also been shown that microRNAs, which control cellular functions by regulating gene expression, are sensitive and specific biomarkers for cell injury [17]. Matton et al. assessed levels of microRNAs in the perfusate and bile of livers undergoing NMP in an attempt to predict injury to hepatocytes and cholangiocytes, respectively [17]. Twelve human donor livers underwent six hours of NMP on the Liver Assist^TM^ device following a period of SCS. There were higher levels of cholangiocyte-derived microRNAs (CD-MiRNAs) in bile and hepatocyte-derived microRNAs (HD-MiRNAs) in perfusate of livers where there was biochemical evidence (high perfusate AST and lactate levels) of hepatocellular injury and poor function. More interestingly, early levels of HD-MiRNA-122 and CD-MiRNA-222 could predict injury after six hours of NMP [17]. This study identified novel markers for graft viability during perfusion that can be detected during the early stages of perfusion although post-transplantation data are not yet available.

Alternatives to whole human liver perfusion

As demonstrated by Table 1, 15 of the 21 studies included experiments involving the ex-vivo perfusion of whole human livers. Huang et al. described a novel technique of split-liver perfusion where after being placed in an ice-cold University of Wisconsin solution, 11 whole livers declined for transplantation were anatomically divided to allow each half to act as its own control [28]. The division occurred to the level of bifurcation of the HA, PV, and bile duct to allow “splitting” into the left and right hepatic lobes. Each lobe was perfused with an acellular perfusate on two separate custom perfusion circuits with cannulation of the left or right lobar HA, PV, and bile duct and the hepatic veins were left open to drain freely. Perfusion was maintained for three hours at a temperature of 20-21°C, which was chosen to allow more functional information than in HMP without the need for oxygen carriers and their attendant costs. They were able to demonstrate that hepatic lobes from a single liver behave similarly when undergoing sub-normothermic perfusion and confirm the hypothesis that this split-liver perfusion technique allows one lobe to serve as an internal control, which obviates the inevitable significant heterogeneity when discarded human liver grafts are used for ex-vivo perfusion studies [28].

A limitation of the above study by Huang et al. is that the liver is more susceptible to IRI due to the prolonged cold ischaemic time incurred as a result of the splitting process on ice [28]. In 2021, Lau et al. were the first group to perform an ex-vivo liver split during NMP using a red-cell-based perfusate and they were able to demonstrate prolonged viability in excess of six days [29]. Furthermore, Thorne et al. have reported a case study where a liver split into a left lateral segment and an extended right lobe was performed during DHOPE and subsequently transplanted into recipients at two different centres [30]. At the six-month follow-up, both patients were well with functioning grafts. These studies demonstrate the versatility of ex-vivo human liver perfusion and the benefits of perfusing sub-optimally sized or partial liver grafts with the increasing demand for donor organs and the finite availability of whole organs.

Schreiter et al. perfused normal human liver tissue sections obtained following partial hepatectomy for malignancy [15]. The resected specimen was forwarded to the pathology department, which identified and removed a section of healthy tissue that was transported to the research team and stored at 4°C until further processing. A limitation of particular note is that the warm and cold ischaemia times are not specified. This is particularly important since warm ischaemia is likely to begin intra-operatively when the inflow to the resected segments is compromised during hepatectomy if inflow occlusion is employed. Furthermore, since there is a significant time interval between the hepatectomy and the heparin flush prior to perfusion, it is likely that microthrombi may already have formed, increasing the risk of IRI. The group also describes perfusion of the tissue sections being performed via four “suitable vessels” that were cannulated. This is unlikely to accurately reflect hepatic vascular anatomy and the details of the branches cannulated are unclear. Although this study demonstrates a huge step towards more ethical and translational research where the human livers response to drug toxicity can be examined, a more anatomical model such as the use of an isolated hepatic segment for perfusion is more likely to produce results as closely analogous to whole-organ perfusion as possible.

## Conclusions

This review summarises the recently published data describing the results of ex-vivo perfusion experiments on the human liver with the majority of studies being performed in the context of liver allotransplantation. The benefits of HMP, NMP, HOPE/DHOPE, and COR are being increasingly explored in experiments and clinical trials, and work continues to ascertain the optimal preservation technique for liver grafts prior to transplantation. There are also a number of studies exploring novel clinical markers of liver viability, including metabolic profiling and microRNA analysis. We have described the alternatives to whole liver perfusion, describing perfusion of the split liver. The studies explored surgical “splitting” of the liver on ice, during NMP and also during HOPE. In addition to split-liver perfusion, one study performed ex-vivo perfusion of a tissue section taken following partial hepatectomy to study acetaminophen toxicity and surprisingly this was the only study using perfused human liver tissue for experimentation of drug toxicity or disease.

There is clearly a need for further studies examining the whole range of human liver models for translational experimental research to provide an important bridge between cell culture and clinical studies. The obvious potential of the various models employed to date together with the increasing consideration of new techniques using tissue slices and possibly segmental sections now offers research groups new and very promising ethical ex-vivo models to examine a variety of issues, including preservation protocols, liver metabolism, pharmacokinetics, drug development, and drug toxicity.
